# Efficacy and Safety of Extracorporeal Shock Wave Lithotripsy (ESWL) in Patients With Infected Ureterohydronephrosis Due to Ureteral Stones Following Double-J Catheter Insertion

**DOI:** 10.7759/cureus.51742

**Published:** 2024-01-06

**Authors:** Alexandra Carina Bandac, Anca Irina Ristescu, Cristian Radu Costache, Razvan Lucian Bobeica, Theodor Florin Pantilimonescu, Pavel Onofrei, Viorel Dragos Radu

**Affiliations:** 1 Urology, C.I. Parhon University Hospital, Iasi, ROU; 2 Anaesthesiology and Intensive Care, Grigore T. Popa University of Medicine and Pharmacy, Iasi, ROU; 3 Urology, Grigore T. Popa University of Medicine and Pharmacy, Iasi, ROU; 4 Morphofunctional Sciences II, Grigore T. Popa University of Medicine and Pharmacy, Iasi, ROU; 5 Urology, Elytis Hope Hospital, Iasi, ROU

**Keywords:** infected ureterohydronephrosis, double-j catheter related symptoms, double-j catheter, ureteral stones, eswl

## Abstract

Introduction: Double-J ureteral catheters in patients with ureteral lithiasis undergoing extracorporeal shockwave lithotripsy (ESWL) procedures reduce the efficacy of the procedure or have no effect on the stone-free rate. However, the effect of double-J catheters on the patients in whom they were inserted for infected hydronephrosis is not known. The aim of our study was to evaluate the efficacy and safety of the ESWL procedure in patients with ureteral lithiasis and double-J catheters previously inserted for infected hydronephrosis.

Method: We conducted a comparative case-control, match-paired study in a group of patients with ureteral lithiasis treated by ESWL from January 1, 2018, to March 1, 2023, who were divided into two groups according to the presence of the double-J catheter. For each patient with the double-J catheter from the study group, we selected one patient for the control group without the double-J catheter and matched them in terms of size, location of stones, and body mass index (BMI). We analyzed the stone-free rate and complications that occurred in the two groups.

Results: Forty patients with ureteral lithiasis and a double-J catheter inserted for infected hydronephrosis were enrolled in the study group. The control group included 40 patients with ureteral stones without double-J catheters. The patients in the two groups were predominantly men with stones located in the lumbar region and on the right side and with a BMI between 25 and 30 kg/m^2^. The stones had an average size of 0.9+/-0.12mm and 0.89+/-0.15mm, respectively (p=0.624). There was no statistically significant difference in stone-free rate between the two groups after the first session of ESWL (47.5% vs. 52.5%, p=0.502), the second (70% vs. 75%, p = 0.616), and the third session (85% vs. 87.5%, p=0.761). The rate of complications was similar in both groups (7.5% vs. 5%, p=0.761).

Conclusions: The presence of double-J catheters inserted in patients with ureteral stones who underwent ESWL for infected hydronephrosis does not affect the stone-free rate of the procedure or the complication rate. The procedure of ESWL in patients with ureteral lithiasis and double-J catheters inserted for infected hydronephrosis is a safe and efficient method that can be recommended as an initial treatment alongside retrograde ureteroscopy.

## Introduction

Extracorporeal shockwave lithotripsy (ESWL), together with retrograde ureteroscopy, is an established method in the treatment of ureteral stones [1]. In the hope of increasing the efficacy of ESWL in these patients, a ureteral catheterization has been proposed prior to the procedure. Studies have shown that pre-procedural stenting has no advantage in terms of stone fragmentation efficiency [2-4]. Some studies even show a decrease in the efficacy of ESWL, both after one [5-7] and after several sessions [8, 9]. However, in most studies, the double-J catheter was inserted in patients with ureteral lithiasis without a clear indication for urinary diversion in these patients (obstruction with non-functioning kidneys, obstruction with superinfection), with the only purpose of increasing the efficacy of ESWL and possibly reducing morbidity by avoiding episodes of renal colic after the procedure [10-12]. Other studies considered patients with double-J catheters inserted after infected ureterohydronephrosis or with non-functioning kidneys [5] as well as those in whom double-J catheters were placed to reduce morbidity and increase the effectiveness of ESWL [8], resulting in heterogeneous groups, which may ultimately lead to erroneous conclusions regarding the effectiveness and safety of ESWL in patients with double-J catheters. There are no studies so far that include a homogeneous group of patients in the case of pre-procedurally stented patients, such as patients who have had stents inserted due to infected ureterohydronephrosis. In addition, studies of stented patients differ in terms of the duration between the insertion of the double-J catheter and the time of ESWL, depending on the reason for which the double-J catheter was inserted [13]. It is known that the maximum efficiency of ESWL is greater for ureteral stones when they are up to 10mm in size [5, 14], with similar stone-free rates in both the lumbar and pelvic regions [15].

To assess the efficacy and safety of ESWL as a treatment for ureteral stones between 5mm and 10mm, we conducted a case-control, match-paired study. We compared patients with infected ureterohydronephrosis, where double-J catheters were inserted, to those without catheters prior to the procedure. The study evaluated the stone-free rate and the incidence of complications in both groups. This comparison aimed to answer the question: Can ESWL be recommended as a primary treatment option for patients with ureteral stones between 5mm and 10mm and a double-J catheter inserted for infected hydronephrosis?

## Materials and methods

We conducted a retrospective study, which included patients admitted to the Elytis Hospital in Iasi, Romania, between January 1, 2018, and March 31, 2023, who underwent extracorporeal shockwave lithotripsy for ureteral lithiasis. The study was approved by the hospital's ethics committee on July 9, 2023 (approval number: 34). We invited all patients to sign a general consent form allowing us to register healthcare data for retrospective studies (laboratory, demographics, therapy, and comorbidities). The study group included all patients with ureteral stones between 5mm and 10mm and double-J ureteral catheters that had been inserted three weeks prior for infected ureterohydronephrosis. All inserted catheters were 7Ch, with lengths between 26cm and 28cm. For cases with double-J syndrome, we indicated tolterodine 2mg, 1tb, twice daily until the catheter's suppression [16]. If suffering persisted, continuation of the ESWL procedure was abandoned, and patients opted for retrograde ureteroscopy. The presence of ureteral calculi and their size were determined by plain renal radiography. All cases were single ureteral stones; we had no patients with multiple ureteral lithiases.

A urine culture was performed in all patients before lithotripsy, which proved the absence of urinary tract infection (UTI). In addition, we waited three weeks from the insertion of the double-J catheter until ESWL was performed to ensure restitutio ad integrum healing of the upper tract UTI. All these patients received prophylactic antibiotic therapy with cefuroxime 0.5g, a single dose before the procedure, in view of the increased risk of infection in patients with a double-J catheter [17].

After the ESWL sessions, tamsulosin was not recommended, as in other studies [18], since we have no studies related to its use in patients with double-J catheters. To create the control group, we selected, in chronological order and at a 1:1 ratio, any patient with ureteral lithiasis between 5mm and 10mm, without a double-J catheter, hospitalized during the same period. Each patient in the control group was matched with a case from the study group based on body mass index (BMI) categories: below 18.5-24.9 kg/m^2^ (normal), between 25-29.9 kg/m^2^ (overweight), and over 30 kg/m^2^ (obese), as well as the location and size of the stone. This approach allowed us to form two groups of homogeneous patients regarding the size, location of the stones, and BMI, characteristics known from previous studies [19, 20] as predictors of treatment outcomes.

**Figure 1:**
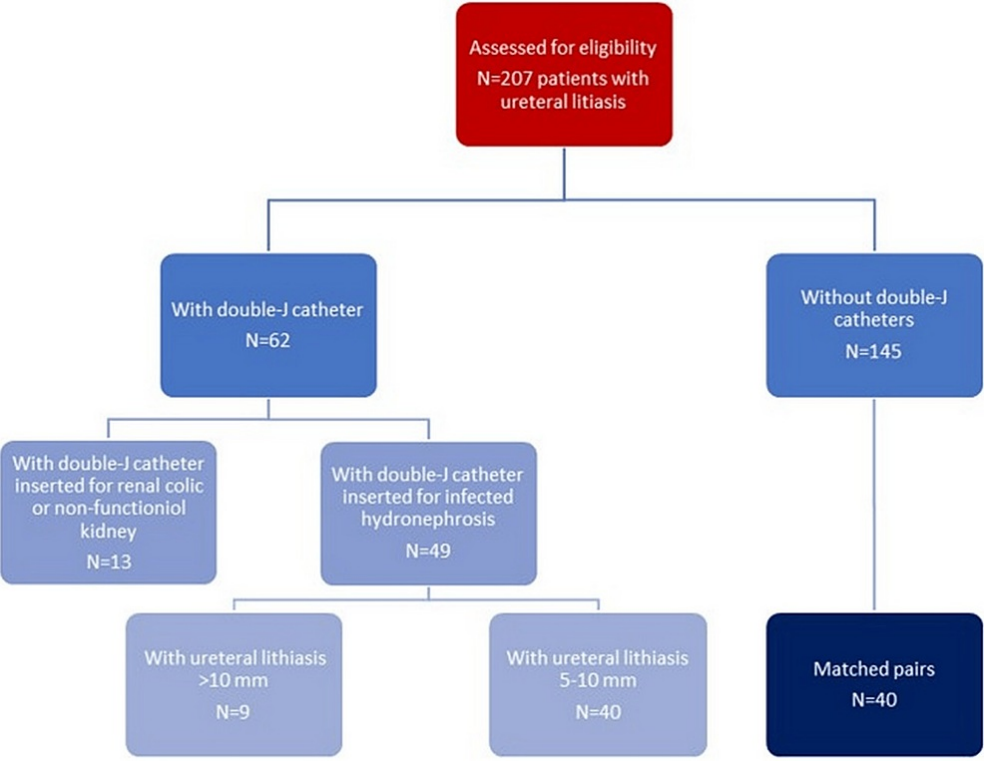
Flowchart for selecting patients for the study

The exclusion criteria were all calculi larger than 10mm, radiolucent calculi objectified by ultrasound or computed tomography, and patients who could not be followed up after the procedure until the calculi were eliminated. In addition, all patients with ureteral calculi and infected hydronephrosis that had migrated into the renal pelvic system after insertion of the double-J catheter were excluded from the study. Figure 1 shows the flowchart for selecting patients for the study.

Extracorporeal shockwave lithotripsy was performed on all patients using the Siemens Sonolith device (Siemens, Nürnberg, Germany). The ramping method was used, whereby the maximum intensity was reached at 1,000-1,200 shock waves, and a number of 2,000-3,000 shock waves were administered. The intensity of the waves was gradually increased from 0.1 per shock wave to a maximum of two. We did not use any anesthetic methods, as the maximum intensity of the shock waves was limited by the occurrence of pain. After the procedure, two weeks in the case of pelvic stones and three to four weeks in the case of lumbar stones, a radiologic control was performed, and a new ESWL session was administered if the fragments were larger than 4mm.

A maximum of three procedures were administered. If fragments of more than 4mm were still found two weeks after the last procedure, retrograde ureteroscopy was proposed to the patient as the ESWL procedure had failed. In cases of fragments up to 4mm, the double-J catheter was suppressed, and the elimination of fragments was checked within two weeks by plain renal X-rays and reno-vesical ultrasound. We defined stone-free rate if no radiopaque fragments were visible after extraction of the double-J catheter in the study group or after the last ESWL session in the control group (two weeks later), and also if ultrasound showed no dilatation of the pyelocaliceal system. All patients with remaining fragments up to 4mm from both groups eliminated the stones spontaneously.

We collected demographic data and diagnostic information from the two groups, including age, gender, BMI, location of stones, and average size of stones. We compared ESWL parameters, specifically the number of sessions required to fragment the stones, the total number of shock waves, the average and maximum intensity of the shock waves, and the total energy required to fragment the stones. Additionally, we analyzed the percentage of stone-free rate in patients after the first, second, and third sessions of ESWL, as well as the occurrence of complications such as double-J stent syndrome, urinary tract infections, febrile syndrome, and renal colic after the procedure.

To avoid possible bias, we only analyzed the procedures performed by two urologists who used the same technique of shock wave application and the same protocol for diagnosis and patient follow-up as previously described.

The number of patients in the study group was limited by the inclusion criteria, consisting mainly of patients in our clinic with ureteral lithiasis but without urinary superinfection and thus lacking the indication for double-J catheter insertion. For the control group, we maintained a 1:1 ratio. Since the design of our paired study reduced the number of eligible patients, and although the size of the two groups may not allow the highlighting of small, statistically significant differences in the stone-free rate, such differences hold no clinical relevance without impacting therapeutic decisions.

Statistical methods

Quantitative variables were described by the mean and standard deviation, and qualitative variables were described by percentages. After testing our data for normality using the Kolmogorov-Smirnov test, we compared quantitative variables using the Student's t-test for normally distributed data and the Mann-Whitney U test for non-normally distributed data. Qualitative variables were compared using the Z-test for proportions. We considered statistically significant differences at p <0.05. The odds ratio (OR) was calculated for the stone-free rates in the two groups after each ESWL session, together with the corresponding confidence intervals.

## Results

During the mentioned study period, 207 patients with ureteral lithiasis were enrolled, and ESWL was used as the primary treatment method, with the procedures performed by two urologists. Among them, 62 patients had previously inserted double-J catheters, while 145 had no double-J catheters. Among the 62 patients with double-J stents, in 49 cases, the catheters had been inserted three to four weeks earlier due to infected ureterohydronephrosis. Out of this group, 40 patients had ureteral stones between 5mm and 10mm, constituting the study group. To form the control group, we included 40 patients without double-J catheters, categorized according to the study methodology. The demographic characteristics and diagnostic information of the two groups are presented in Table 1.

**Table 1:** The characteristics of the two groups *p-value for the Student's t-test; **p-value for Mann-Whitney U test; Nr: number

		Double-J catheter group (n=40)	Non-double-J catheter group (n=40)	p-value for the Z test
Age, mean ± SD (median/limits)		41.00±11.00 (41/22-68)	51.60±16.04 (52/26-84)	0.001*
Gender (Nr., %)	Male	23 (57.5%)	22 (55.0%)	0.818
	Female	17 (42.5%)	18 (45.0%)	0.818
BMI (kg/m^2^)	<25	2 (5.0%)	2 (5.0%)	1.000
	25-30	32 (80.0%)	32 (80.0%)	1.000
	>30	6 (15.0%)	6 (15.0%)	1.000
Size of the calculi (mm), mean±SD (median/limits)		0.90±0.12 (0.9/0.6-1.0)	0.89±0.15 (0.9/0.4-1.0)	0.818**
Location of the calculi (Nr., %)	Lumbar (Nr., %)	26 (65.0%)	26(65%)	1.000
	Pelvic (Nr., %)	14 (35.0%)	14(35%)	1.000
	Right side (Nr., %)	27 (67.5%)	22 (55.0%)	0.250
	Left side (Nr., %)	13 (32.5%)	18 (45.0%)	0.250

There were no statistically significant differences in terms of gender or BMI. Patients in the group with double-J catheters were younger. There was also no significant difference between the two groups in terms of stone size. In both groups, patients with a BMI between 25 and 30 kg/m^2^ had stones in the lumbar region, and the size of the stones was close to the maximum 10mm interval specified in the study. We had no patients with ureteral iliac stones. The ESWL parameters are listed in Table 2.

**Table 2:** The parameters of ESWL in the two groups *p-value for the Student's t-test; Nr.: number; ESWL: extracorporeal shockwave lithotripsy

		Double-J catheter group (n=40)	Non-double-J catheter group (n=40)	p-value for Mann-Whitney U test
Mean nr. of sessions ± SD (median/limits)		1.50±0.75 (2/1-3)	1.65±0.70 (2/1-3)	0.284
Total nr. of shockwaves, mean±SD (median/limits)		3590±1895 (3590/1500-7900)	3751±1742 (3751/1300-8153)	0.692*
Energy level	Mean, mean±SD (median/limits)	0.67±0.18 (0.66/0.30-1.20)	0.71±0.22 (0.71/0.20-1.30)	0.373
	Maximum, mean±SD (median/limits)	0.75±0.29 (0.75/0.30-1.70)	1.37±0.32 (1.37/0.50-2.00)	0.645
Total delivered energy (Joules), mean±SD (median/limits)		48.8±26.1 (48.7/12.18-109.4)	51.1±25.2 (51.2/7.76-114.84)	0.686*

There was no statistically significant difference between the two groups in terms of the number of ESWL sessions required to fragment the calculi, the total number of shock waves, the maximum energy level, and the total energy applied. The stone-free rate after each ESWL session and the complications that occurred in the two groups are shown in Table 3.

**Table 3:** Stone-free rate and complications incidence in the two groups Nr: number; OR: odds ratio

		Double-J catheter group (n=40)	Non-double-J catheter group (n=40)	OR (CI 95%)	p-value for Z test
Stone-free rate (Nr., %)	After the first session	19 (47.5%)	22 (52.5%)	0.74 (0.30-1.78)	0.502
	After the second session	28 (70%)	30 (75%)	0.77 (0.29-2.08)	0.617
	After the third session	33 (82.5%)	34 (85%)	0.83 (0.25-2.73)	0.764
Complications (Nr., %)	Reflux nephropathy	1 (2.5%)	0 (0%)	-	0.312
	JJ stent syndrome	2 (5.0%)	0 (0%)	-	0.152
	Renal colic	0 (0%)	1 (2.5%)	-	0.312
	Infected hydronephrosis	0 (0%)	1 (2.5%)	-	0.312
Total nr. of complications (Nr., %)		3 (7.5%)	2 (5.0%)	1.54 (0.24-9.75)	0.645

The stone-free rate was achieved in 19 patients (47.5%) in the study group and 22 patients (52.5%) in the control group without statistical differences. After the second and third sessions, the stone-free rate rose in both groups without significant statistical differences. The complication rate was reduced in both groups, with no statistically significant differences. There were no significant statistical differences in the stone-free rate after the sessions between the two groups. Reflux pyelonephritis and double-J syndrome occurred in the double-J group, while post-procedural renal colic and infected hydronephrosis occurred in the non-stented group.

**Figure 2:**
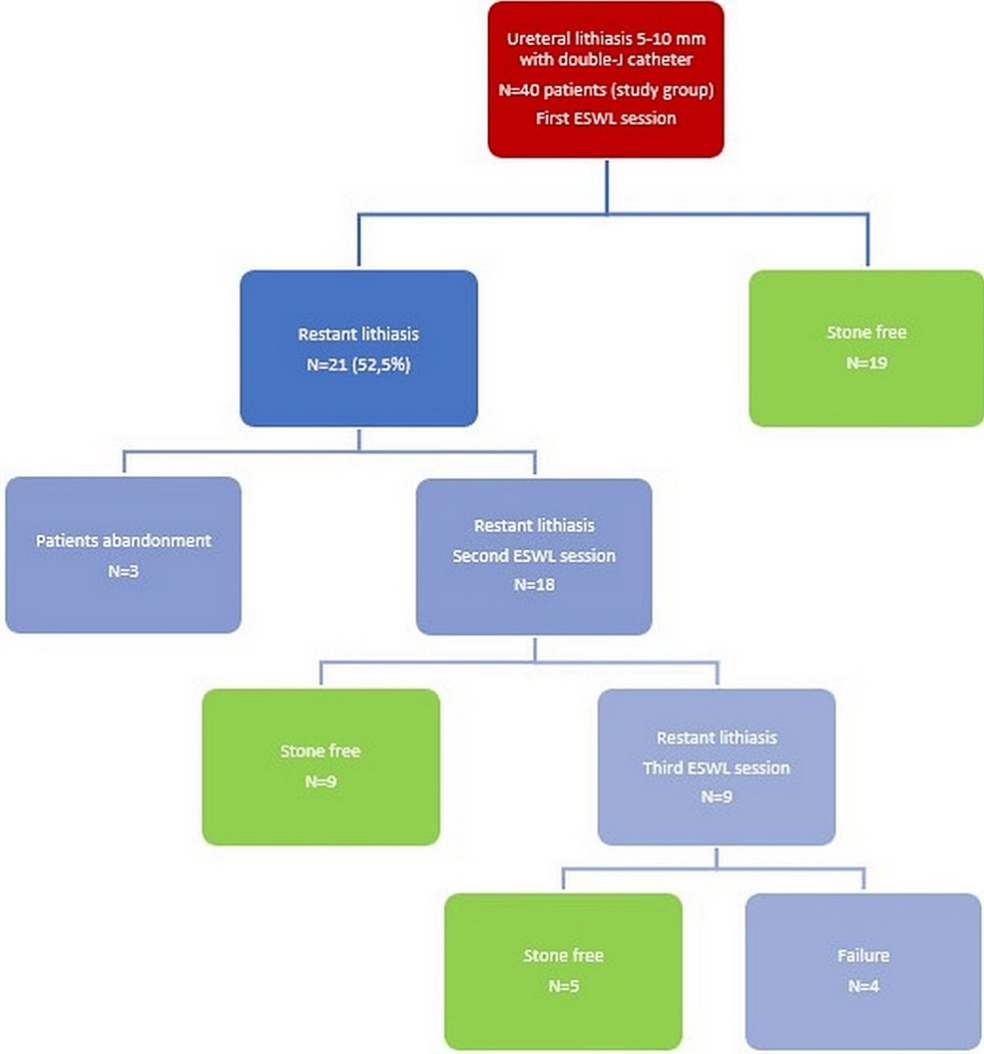
Treatment applied in the study group ESWL: extracorporeal shockwave lithotripsy

**Figure 3:**
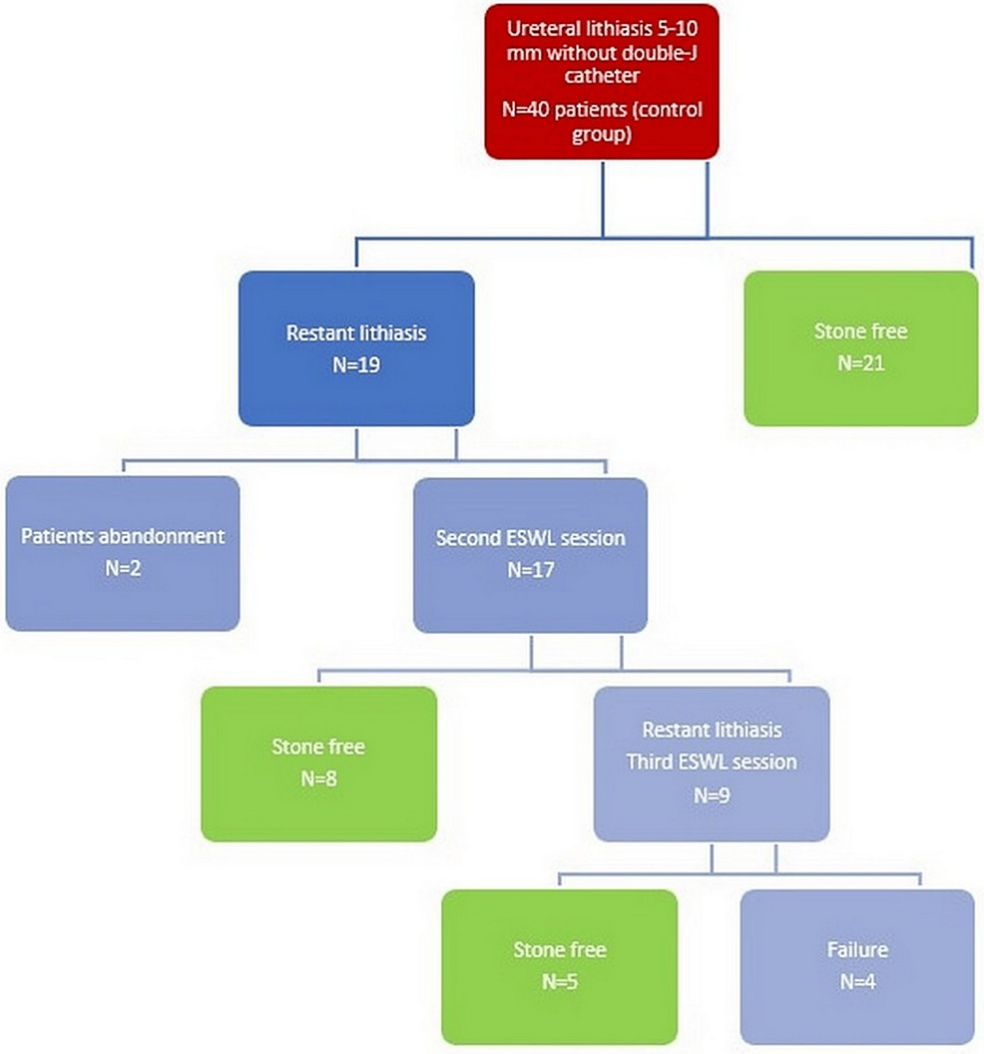
Treatment applied in the control group ESWL: extracorporeal shockwave lithotripsy

Below, we present the flow chart of the treatment applied in both groups (Figures 2-3).

## Discussion

Extracorporeal shockwave lithotripsy has similar efficacy rates in achieving stone-free status regardless of the presence of double-J catheters in patients with ureteral stones between 5mm and 10mm. The complication rate was reduced in both groups. The number was limited by the inclusion criteria of the study, excluding those with double-J catheters inserted due to ureteral obstruction without superinfection. We do not routinely use double-J catheters in our clinic to increase the efficiency of ESWL.

Another limitation would be the fact that we only considered the length of the calculi and not all dimensions, which could influence the stone-free rate [21]. However, the differences in the width of the calculus up to 10mm are small, so we assume that they only marginally influenced our study. The advantage of the study was that it was a match-pair procedure, which eliminated many potential error factors. Also, the creation of a homogeneous group consisting only of patients with double-J catheters inserted for infected hydronephrosis is another advantage, as there is no similar study in the literature to our knowledge.

The reason for previous studies related to the presence of double-J catheters in patients with single ureteral lithiasis was the presence of certain advantages or disadvantages of double-J catheters in terms of increasing or decreasing the stone-free rate in patients with ureteral stones.

The advantages of the presence of double-J catheters would be the creation of an expansion chamber for the fragments from ESWL [13] and the passive dilatation of the ureter, which helps eliminate lithiasis fragments [22]. Meanwhile, the disadvantages would be that the double-J catheter interferes with the shock waves, reducing the effectiveness of ESWL [13] and favoring the occurrence of ureteral edema, which reduces peristalsis and affects the elimination of lithiasis fragments [23]. Our study has shown that there are other favorable and unfavorable factors that impact the efficacy of ESWL. The presence of double-J catheters eliminated the post-procedural incidence of renal colic and febrile syndromes secondary to infected hydronephrosis, which may necessitate the interruption of ESWL sessions and thus reduce the overall stone-free rate. On the other hand, the presence of double-J stents brought new complications, such as double-J syndrome [24-25] and reflux pyelonephritis, which required the interruption of ESWL sessions and thus reduced the overall stone-free rate.

Due to the nature of our study, there were no differences between the groups in terms of the size of the stone, its location, or BMI grade, all known variability factors that may influence the efficacy of ESWL. Most of the patients in the two groups were overweight, predominantly men, and the stones were located on the right side. They had stones with an average size of almost 9mm, predominantly located in the lumbar region. If we exclude these error factors, we can focus on analyzing the influence of the presence of double-J catheters on the efficacy of ESWL in patients with a single ureteral stone smaller than 10mm. The fact that the patients in the group with double-J stents were younger has no influence on the effectiveness of ESWL.

The stone-free rate was over 80% in both groups after the third session, indicating the efficacy of ESWL even in patients with double-J stents inserted for infected hydronephrosis, as well as in other studies showing the lack of influence of the double-J catheter in patients who had undergone ureteroscopy [15, 26]. However, it is worth noting that the stone-free rate after the first session was only about 50% in both groups, even when stones smaller than 10mm were included in the study. This suggests that the energy doses for breaking the stones exceed the dose we administer for one session. However, if we were to increase the dose per session, there would be a risk, especially for lumbar stones, where the shock wave can affect the renal parenchyma and cause renal hematomas, a fearsome complication that must be avoided at all costs, including reducing the efficiency of ESWL in these patients.

The administration of multiple ESWL sessions prolongs the time to achieve stone-free status and thus increases the risk of reflux, pyelonephritis, and double-J syndrome, as emphasized in other studies [13, 27-29]. Reflux pyelonephritis can be a serious complication that can quickly lead to urosepsis in the absence of adequate antibiotic treatment [30]. However, as in other studies [28, 31], the incidence of symptoms associated with double-J catheters in our study was much lower than the nearly 80% reported in other studies [13, 25]. We did not find, as reported in other studies [32], that multiple ESWL sessions are required in patients with double-J stents to achieve the same effect as in patients without stents.

Although no calcifications of the double-J catheters occurred in our study, this risk increases with the duration of wearing the catheter until stone-free status is reached [33, 34]. We had two cases (5%) in the study group in which the second session could not be performed because of symptoms related to the indwelling double-J catheter: macroscopic hematuria and hypogastric pain during urination. We mention that a larger number of patients suffered from double-J syndrome; 11 patients (27.5%), but only two patients (5%) had such severe symptoms that they had to give up continuing the lithotripsy sessions. It is possible that there are a number of patients who suffered from double-J syndrome immediately after insertion of the double-J catheter but did not come for the first session of ESWL. Therefore, it is likely that the percentage of patients with symptoms related to double-J catheters is higher than in our study.

Also, reflux pyelonephritis occurred in a patient in whom the first session had been performed without fragmentation, necessitating the abandonment of the procedure and the indication for retrograde ureteroscopy. Wearing double-J catheters, on the other hand, had certain advantages, as the occurrence of renal colic and infected ureterohydronephrosis could be avoided as complications, which led to the abandonment of the second session in two cases (5%) and necessitated the insertion of the double-J stent and retrograde ureteroscopy. Thus, although the complications of wearing double-J catheters led to the discontinuation of ESWL procedures and thus decreased the stone-free rate, other complications of ureteral lithiasis could be eliminated with a double-J catheter in place, which increased the stone-free rate. Summarizing the pros and cons, we find that the presence of double-J stents had no effect on the stone-free rate in patients with ureteral lithiasis less than 10mm in diameter.

The main limitations of our study are its retrospective nature and the relatively small number of patients. Further prospective studies on larger patient cohorts are needed to validate our findings.

## Conclusions

Our study has shown that ESWL is an efficient and safe method in patients with lumbar ureteral stones of less than 10mm who have a double-J stent inserted for infected ureterohydronephrosis and is a viable alternative to retrograde ureteroscopy. We believe that it can be proposed as the first option for ESWL in these patients, especially when patients are hesitant to accept the idea of a surgical procedure requiring local or even general anesthesia.
